# Spiritualism Pure and Simple

**Published:** 1892-07

**Authors:** 


### SPIRITUALISM PURE AND SIMPLE.

The following eloquent extract is from a memorial sermon delivered in Brooklyn tabernacle by Dr. Talmage.

Had this noted divine confessed to the light which comes with the gift of much despised Spiritualism he could not have done better :

But we must not detain the two garlands any longer from the pillows of those who for a quarter of a century have been prostrate in dreamless slumber, never oppressed by summer heats or chilled by winter's cold. Both garlands are fragrant. Both have in them the sunshine and shower of this springtime. The colors of both were mixed by Him who mixed the blue of the sky, the gold of the sunset, the green of the grass and the whiteness of the snow crystal. And I do not care which you put over the northern grave and which over the southern grave. Does any one say: "What is the use? None of them will know it; your decoration days both sides Mason and Dixon's line are a great waste of flowers." Ah! I see you have carried too far my idea that praise for the living is better than praise for the departed. Who says that the dead do not know of the flowers? I think they do. The dead are not dead. The body sleeps, but the soul lives and is unhindered. No two cities on earth are in such rapid and constant communication as earth and Heaven, and the two great decoration days of north and south are better known in realms celestial than terrestrial. With what interest we visit the place of our birth and of our boyhood or girlhood days! And have the departed no interest in this world where they were born and reared, and where they suffered and triumphed? My Bible does not positively say so, nor does my catechism teach it, but my common sense declares it. The departed do know, and the bannered processions that marched the earth yesterday to northern graves, and the bannered processions that marched a month ago to southern graves, were accompanied by two grander though invisible processions that walked the air, processions of the ascended, processions of the martyred, processions of the sainted; and they heard the anthems of the churches and the salvo of the batteries, and they stooped down to breathe the incense of the flowers. These August throngs gathered this morning in these pews and aisles and corridors and galleries are insignificant compared with the mightier throngs of Heaven who mingle in this service which we render to God and our country while we twist the two garlands. Hail spirits multitudinous! Hail spirits blest! Hail martyred ones, come down from the King's palaces! How glad we are that you have come back again. Take this kiss of welcome and these garlands of remembrance, ye who languished in hospitals or went down under the thunders and the lightnings of Fredericksburg and Cold Harbor and Murfreesboro and Corinth and Yorktown and above the clouds on Lookout Mountain.

